# The role of empathy and psychological need satisfaction in pharmacy students’ burnout and well-being

**DOI:** 10.1186/s12909-019-1477-2

**Published:** 2019-02-04

**Authors:** Eun Cho, Soohyun Jeon

**Affiliations:** 1 Sookmyung Women`s University, College of Pharmacy, Cheongpa-ro 47-gil 100, Yongsan-gu, Seoul, 04310 South Korea; 2 Sheikh Saud bin Saqr Al Qasimi Foundation for Policy Research, P.O. Box 12050, Ras Al Khaimah, United Arab Emirates

**Keywords:** Pharmacy student, Empathy, Psychological needs, Burnout, Psychological well-being, Korea

## Abstract

**Background:**

The purpose of this study was to examine the relationship between Korean pharmacy students’ empathy and psychological need satisfaction and their levels of burnout and psychological well-being, using structural equation modeling.

**Methods:**

The participants were 452 pharmacy students from five South Korean universities. The Jefferson Scale of Empathy (Health Professions Students version), the Activity-Feeling States Scale, and the Maslach Burnout Inventory-Student Survey were used to assess empathy, psychological need satisfaction, and burnout, respectively. Psychological well-being was measured with the Mood Rating Scale, Self-Esteem Scale, and Satisfaction With Life Scale. The fits of the measurement and structural regression (SR) models with data on the four variables were evaluated using the Tucker-Lewis index (TLI), incremental fit index (IFI), comparative fit index (CFI), and root mean-square error of approximation (RMSEA) using AMOS 18.0.

**Results:**

A total of 447 students (98.9%) completed the survey. The measurement model showed adequate fit indices; all hypothesized factor loadings were significant. The proposed SR model also showed an acceptable fit (TLI = 0.92, IFI = 0.94, CFI = 0.94, RMSEA = 0.072); each path was supported except the path from empathy to burnout (β = 0.005). Empathy was positively associated with psychological well-being (β = 0.18). Perceived satisfaction of psychological needs was positively related to psychological well-being (β = 0.59), but strongly and negatively related to burnout (β = − 0.71). The model explained 50 and 44% of variances in burnout and psychological well-being, respectively.

**Conclusions:**

Pharmacy students’ empathy and psychological needs should be considered in pharmacy education systems to promote psychological adjustment.

## Background

Since the Korean pharmacy education program was transformed into a 2-year pre-Pharm + 4-year PharmD system in 2011, it has been found to exacerbate academic stress and burdens for pharmacy students by requiring them to spend two additional years or more preparing for the pharmacy entrance examination. In the existing literature, pharmacy coursework was regarded as a major source of stress for pharmacy students in a nationwide U.S. sample [1]. Another study reported that individuals entering the health care professions, like nursing and pharmacy, tend to be vulnerable to stress in the U.K. and Japan [2]. While pharmacy professionals experience moderate or high levels of job-related stress and burnout similar to those of other health care professionals [3–6], ,pharmacy students’ stress burden seems to be higher than that of practicing pharmacists, possibly because they are undergoing considerable changes in progressing from being college students to health professionals [7, 8].

Health professionals’ burnout and psychological well-being are important in that they affect the quality of life of the professionals themselves as well as the quality of their patient care [9, 10]. Burnout serves as a negative predictor of patient care outcomes and work satisfaction in the context of health care professions and education [11–14]. For example, burnout in physicians is negatively associated with patient satisfaction and quality of patient care, while it is positively related to the occurrence of patient safety incidents [11, 15]. Distress in medical residents has been found to be associated with perceived medical errors [13]. Also, pharmacy students’ burnout is associated with lower academic satisfaction [16]. The negative influence of burnout has been supported not only for health professionals but also for other professionals. Previous research has shown that burnout is positively linked to health problems and employee turnover rates, and negatively linked to job satisfaction and organizational commitment [9, 17, 18]. A longitudinal study indicated that burnout in employees mediates the relationship between work overload and psychological ill-health symptoms, in a process that caused impairment to the employees’ well-being [17].

Moreover, psychological well-being serves as a positive predictor of an individual’s optimal functioning, and quality of life [19, 20]. Psychological well-being includes individuals’ cognitive and emotional evaluations of their lives in terms of perceived life satisfaction, the presence of positive emotions, and the absence of negative emotions [21]. Several studies have reported that physicians’ levels of psychological well-being affect perceived medical errors and patients’ outcomes via their influence on medical errors or serious mistakes [10, 12, 13].

Given the significance of burnout and psychological well-being for people’s ability to function optimally at work and their quality of life, as well as their patient outcomes in the case of health professionals, it is of particular importance to examine what factors affect burnout and psychological well-being. Previous research has suggested that empathy serves as an important predictor of burnout and psychological well-being in the health care professions and education via findings supporting a negative association between empathy and burnout as well as a positive relationship between empathy and well-being [22, 23]. For example, medical students with high levels of empathy exhibit lower levels of burnout and distress [22, 24], and physicians’ levels of empathy are also negatively related to burnout [25]. In addition, medical students’ and emergency nurses’ high levels of empathy were linked to greater psychological well-being [24, 26]. Considering the positive effects of empathy on health professionals’ and students’ burnout and psychological well-being, it is essential for pharmacy educators to investigate the relationships between these variables in the context of pharmacy education.

Although there are relatively few studies in health care contexts, previous studies in other areas (e.g., education, sports, and business) have found that the satisfaction of basic psychological needs is a key factor affecting psychological well-being and burnout [27–29]. In particular, according to self-determination theory, there are three basic psychological needs that are fundamental inner sources of psychological well-being: autonomy, competence, and relatedness [30, 31]. Autonomy refers to one’s inherent need to experience a sense of choice and to self-endorse one’s own behavior [28, 32, 33]. People need to feel that they have voluntarily initiated their behavior and willingly carried it out by themselves rather than having it imposed by an outside source [28, 34, 35]. Competence represents the need to interact with the environment effectively and to engage in challenging tasks to extend one’s capabilities [28, 32, 36]. Finally, relatedness refers to the need to feel securely connected and cared for in an intimate relationship [32, 34, 37].

Previous research has shown that the fulfillment of these three needs has a positive effect on people’s well-being in various domains [28, 30, 38, 39]. For example, employees—including health sector workers—with a greater sense of psychological need satisfaction were more likely to show lower burnout and higher vigor [27, 33].{Van den Broeck, 2008 #4502} In sports and physical education settings, athletes’ psychological need satisfaction was negatively associated with their burnout [40, 41], while it was positively related to subjective vitality [38, 40]. Medical students with greater psychological need satisfaction were more likely to experience lower academic burnout [42], and nurses’ higher levels of need satisfaction were associated with greater job-related affective well-being [43]. Pharmacists’ need frustration was related to low vitality [44], and Korean students’ greater sense of psychological needs were linked to higher levels of positive affect but to lower levels of negative effect [45, 46].

Furthermore, a previous study reported that need satisfaction both partly and fully mediates the relationship between work-related pressure, such as job demands, job stress, and individuals’ burnout, and low psychological well-being [27, 47]. Even though the influence of Korean pharmacy students’ psychological need satisfaction on burnout and psychological well-being has not yet been explored, the results of previous studies suggest potential positive effects of psychological need satisfaction on psychological adjustment, such as lower burnout and greater psychological well-being, in pharmacy education settings.

Currently, even after completing a 2-year pre-Pharm program, the new 4-year PharmD students are still required to complete all coursework from the former 4-year pharmacy program, in addition to a 1-year practicing externship during the 4 years of the Korean program. Although the extension of pharmacy education by 2 years aimed to expand pharmacists’ role to better provide patient-centered care with professional accountability, the additional years of study and increased volume and level of difficulty of the new academic requirements are assumed to increase burnout and decrease psychological well-being, especially during the course of PharmD academic qualification. Therefore, it might be valuable to understand how pharmacy students’ perceptions of psychological need satisfaction relate to their psychological well-being and burnout, which serve as important predictors of future pharmacists’ performance and patient outcomes.

Although there are some studies that have measured and reported the burnout level or depression of medical students in Korea [48, 49], and the relations of medical students’ psychological need satisfaction to their engagement [50], to the best of the authors’ knowledge, there are no reported studies on how empathy and psychological need satisfaction are associated with psychological well-being and burnout for Korean pharmacy students.

**Fig. 1:**
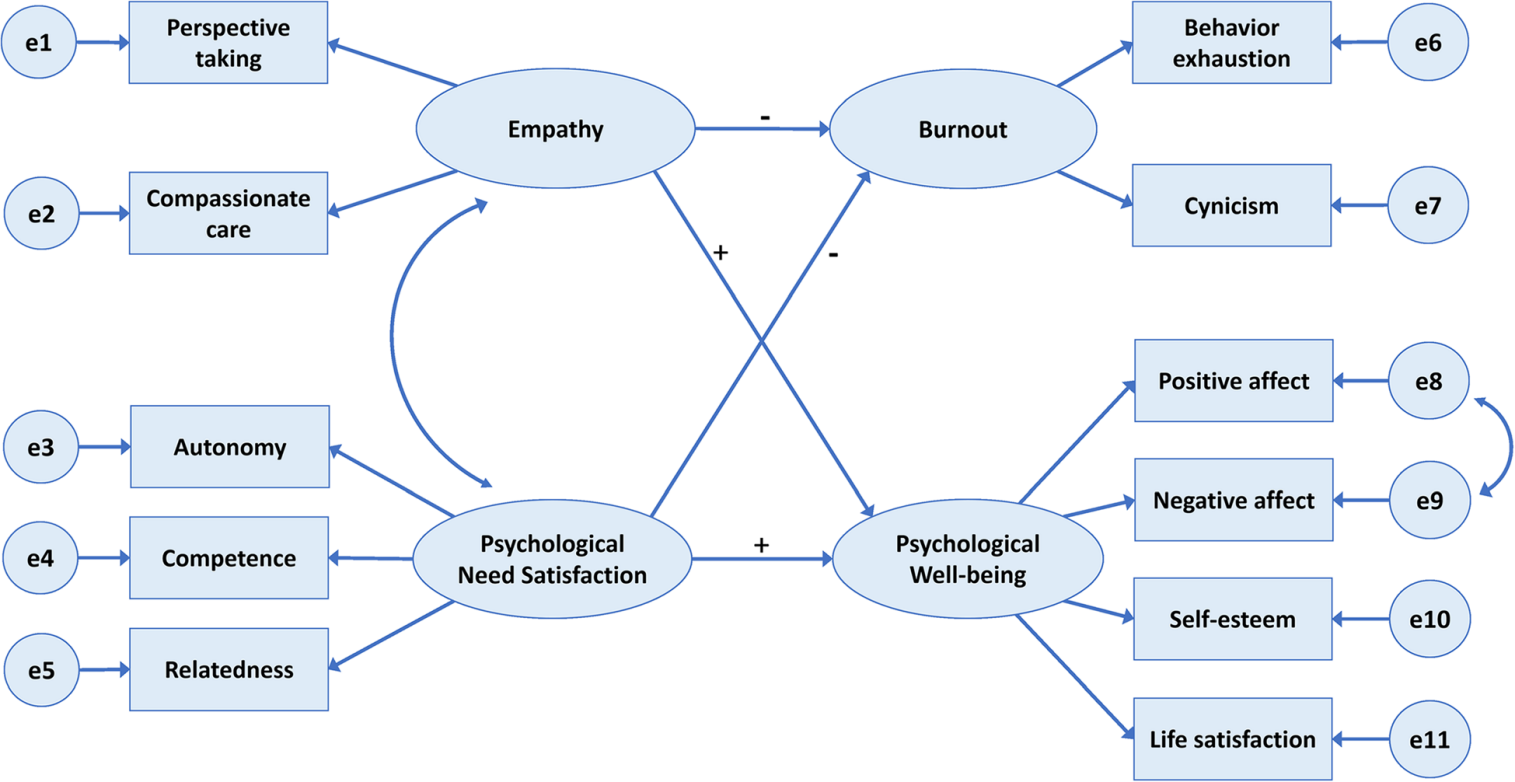
The hypothesized model of burnout and psychological well-being in Korean pharmacy students

Thus, this study was conducted to examine how Korean pharmacy students’ empathy and psychological need satisfaction relate to their academic burnout and psychological well-being. The general purpose of the study was to test the proposed model shown in Fig. 1, as we hypothesized that the proposed model of burnout and psychological well-being would fit the data well for a sample of Korean pharmacy students.^1^ Based on the relationships supported by previous research, we established the following specific hypotheses: Korean pharmacy students’ perceptions of the satisfaction of their psychological needs would relate positively to psychological well-being but negatively to burnout; and students’ empathy would be negatively associated with burnout but positively associated with psychological well-being.

## Methods

### Participants and procedure

We surveyed pharmacy students from five universities with relatively large enrollment in South Korea by means of convenience sampling. The five universities consisted of two national co-ed universities, two private women’s universities, and one private co-ed university, all located in larger Korean cities. Indeed, participating universities in this study comprised approximately 20% (*n* = 413 for the entrance quota) of Korea’s current capacity (*n* = 1693 for the entrance quota per year) for pharmacy students.

After approval from the University Institutional Review Board (No. SM-IRB-13-0823-004) and from the faculty member in charge of each participating class, the survey was administered to second or third-year pharmacy students during five mandatory classes and one elective class at the five universities. The number of potential participants was 479, consisting of 70 from University A, 68 from University B, 120 from University C, 95 from University D, and 126 from University E.

The survey was distributed to all students who attended any of these classes on the scheduled survey day. Data were collected consistently across all six classes. All participants were briefly informed of the purpose of the study and assured of voluntary and anonymous participation. They were also told that the survey responses were confidential and would be used for research purposes by researchers only. A gift of a stationery file holder was provided for all survey participants.

### Measures

The survey was designed to measure four study variables: pharmacy students’ empathy, psychological need satisfaction, academic burnout, and psychological well-being. We used the existing Korean-translated versions of the scales because these measures have demonstrated adequate levels of reliability and validity in previous studies [45, 51–53]. All survey items were rated using a 7-point Likert scale ranging from “strongly disagree” to “strongly agree.”

First, we used the Korean translation of the Jefferson Scale of Empathy Health Professions Students version (JSE-HPS) to assess pharmacy students’ empathy. The Korean versions of the Jefferson Scale of Empathy have demonstrated acceptable reliability (αs = 0.69~0.84) and construct and criterion validity among different health professionals (e.g., nurses and physicians) and health profession students (e.g., medical and pharmacy students) [52, 54–56]. The Korean JSE-HPS is a 15-item scale composed of three subscales: perspective taking (9 items; e.g., “Pharmacists should try to think like their patients in order to render better care”), compassionate care (4 items, reverse scored; e.g., “Attention to patients’ emotions is not important in patient interviews”), and standing in the patient’s shoes (2 items, reverse scored; e.g., “Because people are different, it is difficult to see things from patients’ perspectives”). In the present study, the subscale of standing in the patient’s shoes was not used as an indicator of the latent variable of empathy in the final analysis, as the magnitudes of its correlations with the other two subscales of empathy were small (r = 0.12 with perspective taking; r = 0.16 with compassionate care) and it is considered a trivial component of the empathy scale [57]. The internal consistencies in the present investigation were 0.84 for perspective taking and 0.71 for compassionate care.

Second, we used a 13-item Activity-Feeling States scale [58] to assess Korean pharmacy students’ perceived psychological need satisfaction with regard to their school experiences. Evidence of adequate internal consistency (αs = .73~86) and good predictive validity has been found in previous research [45, 59]. This scale is composed of four subscales: autonomy (4 items; e.g., “My pharmacy school life makes me feel I’m doing what I want to be doing”), competence (3 items; e.g., “My pharmacy school life makes me feel my skills are improving”), relatedness (3 items; e.g., “My pharmacy school life makes me feel I belong and the people here care about me”), and tension (3 items; e.g., “My pharmacy school life makes me feel uptight”). However, for this study we used only the three subscales relating to three basic psychological needs: autonomy, competence, and relatedness. We discarded one item from the autonomy scale (“My pharmacy school life makes me feel free”) because its inter-correlations with the other items were relatively low (rs = 0.04–0.34). Internal consistencies in the present study were 0.65 for perceived satisfaction of the need for autonomy, 0.71 for perceived satisfaction of the need for competence, and 0.73 for perceived satisfaction of the need for relatedness.

Third, we assessed students’ burnout using the Korean version of the Maslach Burnout Inventory—Student Survey [53]. This scale is composed of three subscales: emotional exhaustion (5 items; e.g., “I feel emotionally drained from my studies”), cynicism (4 items; “I doubt the significance of my studies”), and professional efficacy (5 items, reverse scored; e.g., “In my opinion, I am a good student”). This scale has shown adequate levels of internal consistency (αs = .77~84) and construct and criterion validity in previous research [53]. In the present study, we excluded the subscale relating to professional efficacy, as it was highly correlated with the competence subscale of the scale used to measure psychological need satisfaction (r = − 0.66), and their inter-correlation was higher than its correlations with the other two burnout subscales. This may be due to overlapping items between these two measures. In the present investigation, the internal consistencies were 0.81 for behavioral exhaustion and 0.81 for cynicism.

Finally, we assessed pharmacy students’ psychological well-being with the following three instruments: (a) the Mood Rating Scale (MRS) [60], (b) the Self-Esteem Scale [61], and (c) the Satisfaction With Life Scale (SWLS) [60]. These indices of psychological well-being have been widely used in prior research with Western as well as non-Western samples [51, 62, 63].

Specifically, we used the nine adjectives from the MRS to measure pharmacy students’ recently experienced positive and negative emotions [60]. The MRS has demonstrated high levels of reliability in previous research (αs = 0.82–0.89) [63–65]. It consists of a 4-item positive affect scale (i.e., “joyful,” “happy,” “pleased,” and “enjoyment/fun”) and a 5-item negative affect scale (i.e., “depressed,” “worried/anxious,” “frustrated,” “angry/hostile,” and “unhappy”). In the present study, internal consistencies for positive affect and negative affect were 0.94 and 0.81, respectively.

We assessed participants’ global self-worth with the 10-item Self-Esteem Scale [61] (e.g., “I feel that I’m a person of worth or at least on an equal plane with others”). This scale has shown adequate internal consistency (αs = 0.75–0.87) and construct validity in past studies [51, 61, 62, 66, 67]. The original scale used a 4-point Likert scale that ranges from “strongly disagree” to “strongly agree.” In the present study, however, a 7-point Likert scale was used for consistency in the questionnaire format, as the rest of the measures have 7-point Likert scales, and the internal consistency of this scale was 0.88.

We also measured students’ general life satisfaction with the 5-item SWLS [60] (e.g., “In most ways, my life is close to my ideal”). In previous research, the SWLS has been used as an important indicator of subjective well-being and has shown acceptable levels of internal consistency (αs = 0.75–0.91) [63, 68–70]. Internal consistency for this scale was 0.81 in the present investigation.

In this study, all measures of psychological well-being were significantly correlated. Positive affect on the MRS, life satisfaction, and self-esteem were positively associated with one another (*r*s ranged from 0.46 to 0.58, *p* < 0.001), while these factors were each negatively associated with negative affect on the MRS (*r*s ranged from − 0.47 to − 0.54, *p* < 0.001).

### Data analysis

To conduct the data analysis, we first assessed the measurement model via confirmatory factor analysis (CFA) and then evaluated the structural regression (SR) model (i.e., the hypothesized model) using the AMOS 18.0 program. The measurement model contained four latent constructs: empathy, psychological need satisfaction, burnout, and psychological well-being. The structural regression model had two exogenous variables (empathy and psychological need satisfaction) and two endogenous variables (burnout and psychological well-being).

The latent construct of empathy was measured using three subscales of the JSE-HPS: perspective taking, compassionate care, and standing in the patient’s shoes. Second, the latent construct of psychological need satisfaction was assessed using the three subscales of the Activity-Feeling States Scale: perceived satisfaction of the need for autonomy, competence, and relatedness. Third, the latent construct of burnout was measured by two subscales of the Maslach Burnout Inventory—Student Survey: behavioral exhaustion and cynicism. Finally, the latent construct of psychological well-being was assessed using four subscales: positive affect, negative affect, self-esteem, and life satisfaction.

Tests for univariate and multivariate normality of the data were performed to check the normality assumption. We used several indices to examine the overall fit of both the measurement model and the structural regression model to the observed data, such as a chi-squared test, the Tucker-Lewis index (TLI), the incremental fit index (IFI), and the comparative fit index (CFI). In addition, the root mean square error of approximation (RMSEA) was used as a standalone index. A nonsignificant chi-squared value indicates that the model adequately describes the sample data [71]. However, the chi-squared statistic is sensitive to sample size. A higher value for the TFI, IFI, and CFI, typically in the 0.90 range, indicates an acceptable fit to the data [72, 73], while a lower RMSEA value indicates a better model. Values of less than 0.05 and 0.08 reflect good and acceptable fit, respectively, and values of equal to or greater than 0.1 suggest a poor fit [73, 74].

## Results

### Descriptive analysis

Data were collected over a period of 2 weeks, and of 479 the potential participants, 452 students attended the classes prearranged for survey administration. All 452 attendees were invited to participate in the survey. Among them, five students failed to complete the survey; therefore, the final sample size was 447. Participants from each university accounted for 15–28% of the entire sample for this study.

As planned, the sample consisted of second-year (*n* = 214, 47.9%) and third-year (*n* = 233, 52.1%) pharmacy students. The average age of the participants was 25 years (standardized deviation [SD] = 2.88). The participants were primarily female (*n* = 366, 81.9%). Generally, the larger proportion of pharmacy students in Korea are female because a few sizable pharmacy schools belong to women’s universities, and because pharmacy school is more popular with female than with male students. For instance, the proportion of new male students in 2017 was 29% for the one co-ed pharmacy school among our sample universities. In terms of school type, the participants were enrolled in co-ed (*n* = 244, 54.6%) and women’s (*n* = 203, 45.4%) universities and in national (*n* = 133, 29.8%) and private (*n* = 314, 70.2%) universities.

Descriptive statistics and internal consistency values for all measures as well as the bivariate correlations of the observed variables used in the hypothesized model are presented in Tables 1 and 2, respectively. As shown in Table 2, the observed variables of each latent variable in the hypothesized model were significantly inter-correlated and the correlations were in the expected directions (r = 0.57 for the two observed variables of empathy; *r*s = 0.37–0.62 for the three variables of psychological need satisfaction; r = 0.49 for the two variables of burnout; and |*r*|s = 0.46–0.58 for the four variables of psychological well-being). The observed variables of empathy and psychological need satisfaction were significantly correlated with the observed variables of burnout and psychological well-being, except for the correlations between two variables of empathy and behavioral exhaustion as one of the burnout indicators (*r*s = − 0.07 and − 0.05, ns).

**Table 1:** Descriptive statistics and values of Cronbach’s alpha for scale scores (*n* = 447)

Construct	Measure	Number of Items	Mean	SD	Skew	Kurtosis	Coefficient α
Empathy	Perspective taking	9	49.19^a^	5.97	−.130	.027	0.84
	Compassionate care	4	23.27^a^	2.91	−.497	.438	0.71
Psychological need satisfaction	Autonomy	3	4.52	1.01	−.004	−.537	0.65
	Competence	3	4.38	1.02	−.079	−.121	0.71
	Relatedness	3	4.92	0.95	−.653	.962	0.73
Burnout	Behavioral exhaustion	5	4.60	1.12	−.690	.186	0.81
	Cynicism	4	3.69	1.26	.023	−.543	0.81
Psychological well-being	Positive affect	4	5.10	1.10	−.432	.071	0.94
	Negative affect	5	2.88	1.08	.540	.190	0.81
	Self-esteem	10	5.39	0.78	−.417	−.224	0.88
	Life satisfaction	5	4.80	1.01	−.090	−.262	0.81

^a^Sum of item scores

**Table 2:** Bivariate correlations among the observed variables

Variable	1	2	3	4	5	6	7	8	9	10	11
1. Perspective taking	–										
2. Compassionate care	0.57^***^	–									
3. Autonomy	0.26^***^	0.15^**^	–								
4. Competence	0.18^***^	0.13^**^	0.62^***^	–							
5. Relatedness	0.17^***^	0.07	0.37^***^	0.41^***^	–						
6. Behavioral exhaustion	−0.07	−0.05	−0.40^***^	−0.39^***^	−0.18^***^	–					
7. Cynicism	−0.12^**^	−0.18^***^	−0.45^***^	− 0.39^***^	− 0.23^***^	0.49^***^	–				
8. Positive affect	0.17^***^	0.17^***^	0.28^***^	0.32^***^	0.36^***^	−0.19^***^	− 0.10^*^	–			
9. Negative affect	−0.14^**^	−0.16^**^	− 0.29^***^	−0.32^***^	− 0.32^***^	0.28^***^	0.18^***^	−0.53^***^	–		
10. Self-esteem	0.19^***^	0.28^***^	0.37^***^	0.45^***^	0.36^***^	−0.24^***^	−0.18^***^	0.46^***^	−0.54^***^	–	
11. Life satisfaction	0.21^***^	0.17^***^	0.40^***^	0.32^***^	0.28^***^	−0.17^***^	−0.17^***^	0.49^***^	−0.47^***^	0.58^***^	–

Note. *N* = 447; Variables 1–2 are indicators for empathy; variables 3–5 are indicators for psychological needs; variables 6–7 are indicators for burnout; variables 8–11 are indicators for psychological well-being

^*^ *p* < .05. ^**^ *p* < .01. ^***^ *p* < .001

### Measurement model

All 11 observed variables met the criteria for univariate normality [75]. That is, the skew values were all less than 3 (− 0.69 to 0.54) with kurtosis values less than 8 (− 0.54 to 0.96). However, Mardia’s test showed that the data significantly deviated from normal multivariate kurtosis (z = 14.38, *p* < 0.001). Because of the non-normality in the multivariate distribution, we assessed the overall model fit using a chi-squared model test statistic of absolute fit with a Bollen-Stine bootstrap-based *p*-value with 2000 resamples [76].

The results of the confirmatory factor analysis showed adequate fit indices, TLI = 0.92, IFI = 0.95, CFI = 0.94, RMSEA = 0.072 (90% CI: 0.059–0.087), even though the chi-squared statistic for the overall model was significant, χ^2^(37, *N* = 447) = 123.50, *p* < 0.001, Bollen-Stine bootstrap *p* < 0.001. All hypothesized factor loadings were in the expected direction and significant (*p* < 0.01). The empathy factor was positively correlated with psychological need satisfaction (r = 0.30, *p* < 0.001) and psychological well-being (r = 0.36, *p* < 0.001), and slightly negatively correlated with burnout (r = − 0.21, *p* < 0.01). The psychological need satisfaction factor was highly positively correlated with psychological well-being (r = 0.66, *p* < 0.001), while it was highly negatively associated with burnout (r = − 0.72, *p* < 0.001). Psychological well-being and burnout were negatively inter-correlated (r = − 0.37, *p* < 0.001).

### Structural regression model

**Fig. 2:**
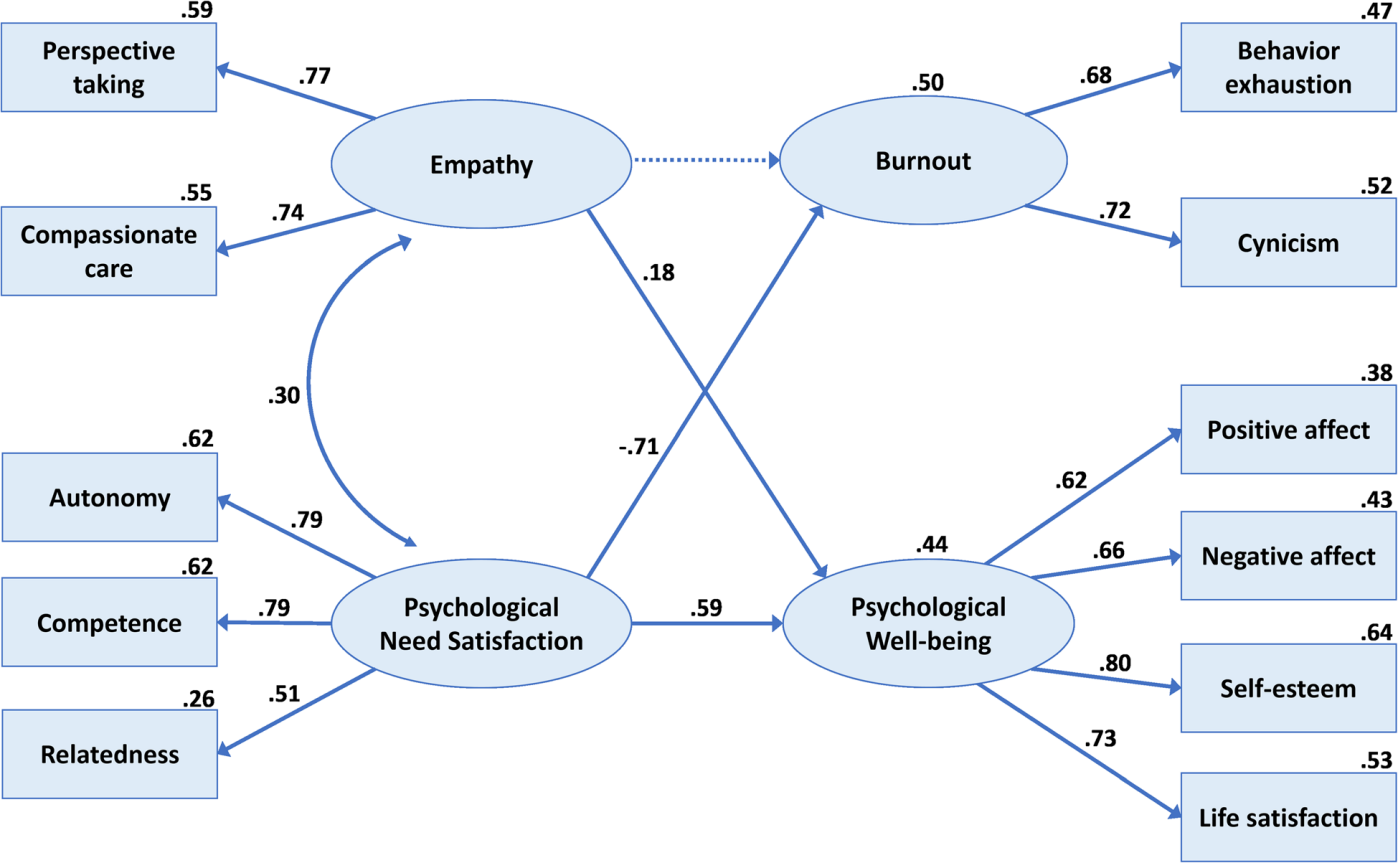
Final model of Korean pharmacy students’ burnout and psychological well-being

The results of the structural equation modeling analysis indicated that the proposed model fit the observed data adequately, TLI = 0.92, IFI = 0.94, CFI = 0.94, RMSEA = 0.073 (90% CI: 0.059–0.087), even though the chi-squared statistic for the overall model was significant, χ^2^(38, *N* = 447) = 128.013, *p* < 0.001, Bollen-Stine bootstrap *p* < 0.001. This analysis showed that each hypothesized path within the model was supported except the path from empathy to burnout (β = 0.005, n.s.). Thus, the fit of the modified model was assessed after eliminating this hypothesized path from the original model. The results revealed that the modified model showed an acceptable fit, TLI = 0.92, IFI = 0.94, CFI = 0.94, RMSEA = 0.072 (90% CI: 0.058–0.086), even though the chi-squared statistic for the overall model was significant, χ^2^(39, *N* = 447) = 128.146, *p* < 0.001, Bollen-Stine bootstrap *p* < 0.001. The result of the chi-squared difference test demonstrated that there was no significant difference between the two models, ∆χ^2^(∆df = 1, N = 447) = 0.133, n.s., which means the overall fit of the modified model was not improved by eliminating the path from empathy to burnout. The standardized path coefficients and the proportion of variance explained by each endogenous variable are presented in Fig. 2.

As shown in Fig. 2, pharmacy students’ perception of their psychological need satisfaction was related strongly and negatively to their level of burnout (β = −.71, *p* < 0.001), and was related positively to their psychological well-being (β = 0.59, p < 0.001). Additionally, students’ empathy was positively associated with their psychological well-being (β = 0.18, *p* < 0.01), but the magnitude of the effect was much smaller than that of the effect relating to psychological need satisfaction. This model explained 50 and 44% of the variances in burnout and psychological well-being, respectively.

## Discussion

In this study, we examined a hypothesized model of Korean PharmD students’ burnout and psychological well-being by exploring the relationships with the levels of empathy and psychological need satisfaction. In the final model, both empathy and psychological need satisfaction were significantly and positively associated with psychological well-being. Psychological need satisfaction was also significantly and negatively related to burnout. However, the path from empathy to burnout was not statistically significant and was therefore eliminated in the final model.

Both the positive relationship between psychological need satisfaction and psychological well-being and the negative relationship between need satisfaction and burnout for pharmacy students are consistent with the results of previous studies in various work settings [28, 29, 33]. Previous studies have reported that psychological need satisfaction negatively related to burnout via factors such as exhaustion, depersonalization, and lack of personal accomplishment for employees; it positively related to psychological well-being [77–79]. The significantly positive relationship between empathy and psychological well-being observed in the current study is also supported by previous studies with medical students [22, 24]. However, the influence of empathy on burnout was not significant in the present study, while the relationships between these two variables have been supported with health care professionals and students in previous research [24, 80].

The present findings provide additional empirical support for the proposition of SDT that perceptions of psychological need satisfaction affect optimal psychological functioning [81]. In addition, the present study supported the functional significance of Korean pharmacy students’ psychological need satisfaction by showing that it accounted for 44% of the variance in students’ psychological well-being with empathy and also for 50% of the variance in burnout, independently. To best of our knowledge, to date there are no studies that examine the role of psychological need satisfaction on pharmacy students’ psychological well-being and burnout. Also, there has been limited research to examine the relationships between empathy and health care professional students’ psychological outcomes [24], compared to research focusing on its relationships with academic and clinical performance or patients’ satisfaction [16, 82–84]. Given the beneficial influences of both empathy and psychological need satisfaction on pharmacy students’ psychological well-being, the results of the present study may contribute to better understanding of the psychological factors that promote pharmacy students’ psychological adjustment, which should be considered important in pharmacy education.

In addition to the aforementioned theoretical implications, the present findings offer practical implications for the prevention of students’ burnout and the enhancement of their psychological well-being. Because the results suggest that students’ psychological need satisfaction is a substantive predictor of their psychological adjustment, and considering the competitive nature of Korean pharmacy education settings, promoting pharmacy students’ psychological need satisfaction may be important in Korean pharmacy education.

Although the environmental factors were not included in the model of the current study, previous research has examined how learning environments either fulfill or thwart students’ sense of autonomy, competence, and relatedness. For example, the autonomy support from instructors was significantly related with the competence dimension in both Asian and Western cultures, and the relation was even stronger for Chinese students, compared with British students [39]. This implies that autonomy-supportive learning environments created by faculty such as professors and instructors should be included in the pharmacy education environment, which in Korean pharmacy schools features competitive and stressful learning environments, to promote a positive and satisfying learning experience for students.

Although the magnitude of the relationship with psychological well-being was relatively small, the independent effect of empathy on psychological well-being was still significant after controlling for the effect of psychological need satisfaction. Also, previous research has supported the positive influences of empathy on various health care outcomes other than psychological well-being and burnout, such as health care professionals’ confidence in patient treatment, quality of interactions with patients, and patient compliance with treatment [82–85]. Taken all together, empathy is a significant element of pharmacy professionalism and education [86–89], and thus pharmacy curricula should be thoughtfully discussed and developed to understand the level of empathy of current students and to incorporate empathy training to strengthen this characteristic. Previous research has shown that pharmacy students’ empathy can be improved by learning activities such as community service learning [90, 91] and learning simulation [92]. However, neither considerable attempts nor other efforts have been afforded to the curriculum design and empathy training course of the PharmD program in Korea.

### Burnout and psychological well-being in pharmacy students

The prevalence of burnout and stress-related mental health problems in physicians and nurses is not a recent development [93, 94]. Burnout is a key indicator that can be used to anticipate mental health problems and to prevent suicide and suicidal ideation in students [53]. Indeed, burnout is related not only to mental but also to physical health. A previous study has reported that it is possible to distinguish health providers in a state of burnout from others by observing their poorer self-rated health, depression, anxiety, sleep disorders, and perceived memory [95].

Especially for health profession students, burnout affects cognition and behavioral responses, which could result in the loss of motivation and perceived depression in the long term [10, 93, 94]. Currently, the reduced graduate school admission rate of pharmacy graduates in Korea might reflect the tendency to low academic achievement, decreased self-efficacy, and reduced motivation. Furthermore, considering that burnout is an important determinant of intention to leave the profession for health providers [94], strategies to reduce or manage burnout need to be developed in both academic and health care institutions to ensure professional commitment in pharmacists and prevent the unnecessary loss of PharmD professionals.

As mentioned, burnout is harmful not only to students’ mental health but also to other individuals, including patients and other health care professionals. For instance, cynicism is likely to negatively affect relationships not only with colleagues but also with patients [11]. In the era of patient-centered care, and considering that an important goal of professional pharmacy education is to generate professional pharmacists who are equipped with this approach, competence in building relationships with patients and other health professionals is critical. However, burnout in pharmacy students and professionals could inhibit the development of their relationship with their colleagues and patients.

While the concept of psychological well-being is complex, higher levels of well-being denote optimal functioning [20]. Previous research has reported that one’s level of subjective well-being is explained not only by personal circumstances but also by cultural variables [19]. For instance, Asian students are more likely to trade off their positive emotions against important future goals such as academic achievement, while the question of whether pursuing cultural value enhances long-term well-being has not yet been answered [19]. In fact, Korean parents’ expectations for and involvement with their children’s education start from early childhood and continue into young adulthood [96]. The aspiration to a professional career is supported by parents and society, and there is keen competition to enter medical or pharmacy school, as this promises a professional position after graduation, which is prized in Korean culture. However, as the competition is severe, there has also been a high threshold for students to enter and study at pharmacy school.

In addition, the efforts made by pharmacy schools to promote students’ mental health may need to be legally regulated. According to the Guidance for Assessment by the Accreditation Council for Pharmacy Education of the U.S.: Accreditation Standards 24, which mentions that “these assessments include measurements of perceived stress in faculty, staff, and students and an evaluation of the potential of stress to have a negative impact on programmatic outcomes and morale,” pharmacy schools are required to identify underlying causes of poor performance by their members [97]. Although the Korean Accreditation Council for Pharmacy Education, established in 2011, has not yet fully established its role or exercised its capacity, it should consider adopting the practice of measuring all stakeholders’ perceived mental health in the process of evaluating pharmacy schools for accreditation. In doing so, students’ performance and mental health could be checked regularly, and they could be supported by the school whenever in need.

### Limitations and future research

We suggest several directions for future research, with consideration given to the limitations of the current study. First, causal relationships between predictors and outcome variables are not warranted because the results are correlational in nature. Therefore, future research needs to conduct a longitudinal or an experimental study in order to clarify the causal paths from pharmacy students’ empathy and satisfaction of psychological needs to their burnout and psychological well-being.

Second, due to a potential model complexity, we used the mean or sum score of each subscale as observed variables of the latent variables in the CFA and SR model rather than individual items. Since three of the latent variables included only two or three observed variables, we tested the total measurement model with the four latent variables instead of testing the measurement model of each latent variable independently. Hence, it is important for future research to validate the measurement model of each measure to ensure good psychometric properties of each scale before examining the relationships among the variables in an SR model.

Third, in future studies, factors increasing pharmacy students’ empathy and psychological need satisfaction may need to be examined. If we consider that one of the goals of pharmacy education is to reduce students’ levels of burnout and promote their psychological well-being, we may want to find specific resources relevant to the satisfaction of psychological needs in order to utilize them for the purpose of decelerating and preventing the sequence of problems leading to burnout and improving psychological well-being. Therefore, future studies might benefit from a more extended model of pharmacy students’ burnout and psychological well-being by adding certain measures of the learning environment to the model. Previous studies have shown that students’ psychological needs are satisfied when their learning environment supports their autonomy and offers optimal structure and involvement [98–100]. To gain a more comprehensive understanding of Korean pharmacy students’ psychological need satisfaction and adjustment in school, it might be important to explore the influences of the learning environment on Korean pharmacy students’ perceived need satisfaction by incorporating these contextual factors into the model. If so, such a finding would give pharmacy educators a practical insight into how to create a healthy learning environment that supports students’ psychological need satisfaction and psychological well-being.

Fourth, in contrast to some of the previous research for other health care professionals [24, 101, 102], this study failed to support the independent effect of students’ empathy on their burnout, as the relationship between these variables was not significant in the model, and the bivariate correlations with two elements of burnout (emotional exhaustion and cynicism) were insignificant or weak. Hence, future research also needs to investigate whether such patterns of associations between empathy and psychological adjustment are replicated in a more representative sample.

The present study included only second and third-year PharmD students from pharmacy schools in large cities. Therefore, it is important for future research to test the generalizability of the hypothesized model to pharmacy students of different years of study or schools. This is the first study to test a model of Korean pharmacy students’ burnout and psychological well-being, and thus it would be valuable to replicate our work in a more representative pharmacy student sample that includes students in different years of study as well as licensed pharmacists. While the relationships between variables are expected to be similar, the roles of influencers could vary according to other aspects of the practice settings of PharmD graduates.

Finally, the development of a program to coach and empower future professionals by anticipating and examining the risk factors for burnout and poor psychological well-being should be initiated. Furthermore, the effects of the implemented interventions on changes in pharmacy students’ burnout and psychological well-being should be explored in future research.

## Conclusions

To our knowledge, this is the first study to identify the effects of empathy and psychological need satisfaction on burnout and psychological well-being in Korean pharmacy education, using structural equation modeling. Pharmacy students’ empathy and psychological needs should be taken into consideration in the Korean pharmacy education system to promote better psychological adjustment. Doing so will help extend the social utility of the PharmD profession, for better results in patient-centered pharmaceutical care.

## Abbreviations

CFI: Comparative fit index
IFI: Incremental fit index
JSE-HPS: Jefferson scale of empathy (Health professions students)
MRS: Mood rating scale
RMSEA: Root mean square error of approximation
SWLS: Satisfaction with life scale
TLI: Tucker-Lewis index

## Footnotes

1 As shown in Figure 1, the disturbance terms of positive affect and negative affect were allowed to be correlated, because these two constructs are interrelated in terms of the shared method of assessing participants’ emotions (i.e., using adjectives), which is not explained by the latent construct of psychological well-being. However, this relationship is not articulated in the study hypotheses because it is not a focus of the present investigation.
